# Transcriptome profiling and in silico docking analysis of phosphine resistance in rice weevil, *Sitophilus oryzae* (Coleoptera: Curculionidae)

**DOI:** 10.1093/jisesa/iead110

**Published:** 2023-12-30

**Authors:** Upasna Selvapandian, Saranya Nallusamy, Sonu Kumar Singh, Jayakanthan Mannu, Varanavasiappan Shanmugam, Caroline Ravikumar, Mohankumar Subbarayalu

**Affiliations:** Department of Plant Biotechnology, Centre for Plant Molecular Biology & Biotechnology, Tamil Nadu Agricultural University, Coimbatore, India; Department of Plant Molecular Biology and Bioinformatics, Centre for Plant Molecular Biology & Biotechnology, Tamil Nadu Agricultural University, Coimbatore, India; Department of Plant Biotechnology, Centre for Plant Molecular Biology & Biotechnology, Tamil Nadu Agricultural University, Coimbatore, India; Department of Plant Molecular Biology and Bioinformatics, Centre for Plant Molecular Biology & Biotechnology, Tamil Nadu Agricultural University, Coimbatore, India; Department of Plant Biotechnology, Centre for Plant Molecular Biology & Biotechnology, Tamil Nadu Agricultural University, Coimbatore, India; Department of Plant Molecular Biology and Bioinformatics, Centre for Plant Molecular Biology & Biotechnology, Tamil Nadu Agricultural University, Coimbatore, India; Department of Plant Biotechnology, Centre for Plant Molecular Biology & Biotechnology, Tamil Nadu Agricultural University, Coimbatore, India

**Keywords:** rice weevil, phosphine resistance, *dld*, mitochondrial gene, detoxification gene

## Abstract

The rice weevil, *Sitophilus oryzae* (Linnaeus, Coleoptera: Curculionidae), is a serious cosmopolitan pest that affects grain in storage and has developed high levels of resistance toward phosphine. In this study, RNA-seq data was used to study the phosphine resistance mechanisms in *S. oryzae*. Resistant and susceptible populations of *S. oryzae* were identified based on phosphine bioassays conducted in 32 populations collected across Tamil Nadu, India. Differential expression of mitochondrial (*COX1, COX2, COX3, ND2, ND3, ATP6*, and *ATP8*) and detoxification genes (*Cyps*, *Gsts*, and *Cbe*) were observed in the resistant and susceptible populations of *S. oryzae*. The previously characterized phosphine resistant gene, *dld* (dihydrolipoamide dehydrogenase) linked to the *rph2* locus, was found to be up-regulated in resistant *S. oryzae* population (ISO-TNAU-RT) treated with phosphine. Also, the genes involved in Tricarboxylic acid (TCA) cycle were significantly down-regulated. In addition, a significant up-regulation in the expression of the antioxidant enzymes superoxide dismutase (2.5×) and catalase (2.1×) in ISO-TNAU-RT populations was recorded. Furthermore, a distinct amino acid substitution, Lysine > Glutamic acid (K141E) was identified in resistant phenotypes. In silico docking studies of both resistant and susceptible DLD protein with phosphine molecule revealed that the amino acid residues involved in the interaction were different. This suggested that the amino acid substitution might lead to structural modifications which reduces the affinity of the target (phosphine). This study provides insight on the various genes, pathways, and functional mechanisms having a significant role in phosphine resistance in *S. oryzae.*

## Introduction

Phosphine (PH_3_) is a fumigant that is used predominantly worldwide to protect storage commodities from insect pests (Bell 2000, Alzahrani and Ebert 2023). Phosphine is a strong reducing agent that disrupts energy metabolism on exposure, primarily the mitochondrial function (Nath et al. 2011). Phosphine is used excessively for the rapid killing of insects and it leaves behind minimal residues on treated products (Oppert et al. 2015). It also has other advantages, such as direct application on commodities, residual-free, low-cost, etc. Also, the availability of alternative fumigants are limited as methyl bromide causes ozone depletion and has been phased out of normal fumigation use (Chaudhry 1997, Kim et al. 2019b). Although the UN’s Food and Agriculture Organization has produced standards for treating stored product pests, including suggested periods and doses based on environmental conditions (https://www.fao.org/home/en/ accessed on 4th April 2021), its frequent application has led to the development of resistance in many storage pests like *Tribolium castaneum* (Herbst, Coleoptera: Tenebrionidae) (Chen et al. 2015, Wakil et al. 2021), *Sitophilus oryzae* (Linnaeus, Coleoptera: Curculionidae) (Daglish et al. 2014), *Rhyzopertha dominica* (Fabricius, Coleoptera: Bostrichidae) (Chen et al. 2015, Wakil et al. 2021), and *Cryptolestes ferrugineus* (Stephens, Coleoptera: Laemophloeidae) (Konemann et al. 2017). High levels of phosphine resistance have been reported in India (Rajendran 1999) and other countries in *S. oryzae*, a serious cosmopolitan pest that affects grains in storage which left uncontrolled results in considerable losses in grain quality and quantity (Cotton 1920). No feasible alternative chemical for phosphine which offers the same spectrum of benefits has been reported.

Phosphine resistance in *S. oryzae* is conferred by 2 phenotypes namely *rph1* and *rph2* (Mau et al. 2012a,b). Cytochrome b5 fatty acid desaturase, an orthologous gene (*Cyt-b5-r*) was found to be associated with the *rph1* locus (Schlipalius et al. 2018) that is a common contributor for phosphine resistance. The *dld* (dihydrolipoamide dehydrogenase) gene that is linked with the *rph2* locus, is an important enzyme in energy metabolism which upon mutations renders phosphine resistance in Coleopteran pests (Schlipalius et al. 2012). The *dld* gene is one of the key mitochondrial enzymes involved with phosphine resistance mechanism through the production of reactive oxygen species (ROS) and the activation of cell-death pathways (Dikalov 2011). Alternatively, phosphine is also known to interrupt antioxidant enzyme activity (Price and Dance 1983). A number of antioxidant enzymes such as catalases, peroxidases, and superoxide dismutase prevents the oxidative stress caused by phosphine through the conversion of H_2_O_2_ to water (Yadav et al. 2020). The phosphine-resistant strain had higher activity of these enzymes than the susceptible strain, implying that increased antioxidant enzyme activity is linked to insect phosphine tolerance. The impact of antioxidant enzymes on phosphine resistance has been reported in the following species: *R. dominica*, *S. granarius*, and *C. ferrugineus* (Price and Dance 1983; Bolter and Chefurka 1990). Hence, understanding the genetics of these resistance phenotypes are essential as it benefits in managing phosphine resistance.

Many diagnostic tools using molecular markers have been developed to identify phosphine resistance that mainly focuses on *dld* genes. In addition, transcriptome analysis has been used for a broader understanding of phosphine resistance mechanisms in *T. castaneum* (Oppert et al. 2015), *R. dominica* (Yang et al. 2018), and *C. ferrugineus* (Chen et al. 2021). Although Kim et al. (2019a) have reported on the proteomic studies on phosphine resistance in *S. oryzae*, it mainly focused on the expression of mitochondrial genes that is involved in phosphine resistance. Here, the present study aimed to give a deeper insight on the transcriptome profile of *S. oryzae* and identify the candidate resistance genes involved in phosphine resistance and study their relative expression levels.

## Materials and Methods

### Insect Populations

About 32 *S. oryzae* populations from the bulk grain storages and grain supply chain across Tamil Nadu, India, were collected. The insect populations used in this study were collected from rice grain samples stored at bulk grain storages and grain supply chain across Tamil Nadu. The sample details are mentioned in Supplementary Fig. S1. The collected insect populations were mass cultured as mentioned by FAO (1975) in disinfected whole wheat grains at room temperature of 30 ± 2 °C and relative humidity of 60 ± 5% for more than 20 generations. Insects were released separately in a 2.5 kg plastic container with the whole wheat and 5.0% brewer’s yeast and placed for oviposition. After oviposition, the emerged grubs were allowed for the development into adults that were used for the study.

### Dose Mortality Response to Phosphine

The resistant and susceptible populations of *S. oryzae* were identified from previous study (Kumar et al. 2022). These populations were fumigated with a series of phosphine concentrations, viz., 0.0010, 0.0050, 0.0075, 0.010, 0.015, 0.04, 0.10, 0.25, 0.5, and 1.0 mg/L for susceptible population. In addition, the resistant population was exposed with the following concentrations of phosphine: 0.05, 0.10, 0.25, 0.50, 1.0, 2.0, 4.0, 6.0, 8.0, and 10.0 mg/L. The FAO recommended concentrations (FAO 1975) for example, 0.04 and 0.25 mg/L for susceptible and resistant populations, respectively, was used to fix these concentrations.

For the phosphine bioassay, the phosphine gas was generated in a phosphine gas generation chamber. A 0.6 g of commercially available aluminum phosphide tablet, wrapped with a muslin cloth was immersed in 1 liter of 5% sulfuric acid water and covered with an inverted funnel. The generated gas was captured in a collecting tube, placed on the inverted funnel. Insects were placed in perforated plastic cups. The cups were placed inside the 2.5 L desiccator and closed with a desiccator lid. The desiccator was tightly sealed by applying silicon grease on the flange of desiccator to avoid the leakage of phosphine gas. The required volume of phosphine gas for each concentration was extracted from the collecting tube using a syringe and injected inside the desiccator through septum. Before injecting the gas, an equal volume of air was removed from the desiccator using a syringe. A separate desiccator was used for each concentration. The volume of the phosphine gas was calculated based on the weight-by-volume basis which was described by FAO method No. 16 (FAO 1975). The fumigation was carried out for 20 h at 25 °C temperature and 60 ± 5% relative humidity beneath the fume cupboard.

A total of 50 adult insects were used for the experiment in triplicates. The insects were exposed to phosphine for 24 h. After the exposure period, the insects were relocated to the recovery room with the culture medium. Mortality was recorded 7 days after exposure. The entire experiment was repeated twice. The median lethal concentrations (LC50 and LC90) with 95% confidence limits were determined by Probit analysis (Finney 1971) using Polo Plus 2.0 software (LeOra Software 2003).

### RNA Sequencing

Total RNA was isolated from phosphine-treated and untreated populations (using both susceptible and resistant) of *S. oryzae* by pooling the adults following the Trizol method (Chomczynski and Mackey 1995). RNA integrity was validated using Nanodrop (Thermo Scientific, USA), Qubit (Thermo Scientific, USA) and Tempstation (Agilent, USA). RNA sequencing libraries were prepared with the Illumina-compatible NEBNext Ultra II Directional RNA library Prep kit (New England BioLabs, MA, USA) at Genotypic Technology Pvt. Ltd., Bangalore, India.

### RNA-seq Analysis

Transcriptome analysis was carried out using OmicsBox software package version 1.3.11 (www.biobam.com/omicsbox). Paired-end reads were pre-processed using FastQC version 0.11.8 for quality assessment. Preprocessing was done by Trimmomatic version 0.38 using a sliding window trimming option to filter and remove low-quality bases (*q* < 30). Reads were filtered using the following default parameters, such as average quality as 25 and minimum length as 36 (Bolger et al. 2014).

The reference genome of *S. oryzae* was downloaded from BioInformatics Platform for Agroecosystem and Arthropods (BIPAA) in GFF format. The preprocessed quality data was aligned with *S. oryzae* reference genome using STAR: Gapped Mapper version 2.7.5 (Dobin et al. 2013). Gene-level quantification was estimated with the “create count table” tool that is based on HTseq package version 0.9.0 (Anders et al. 2015) which counts the number of reads mapped to genomic features. Pairwise differential analysis between the PH_3_—R and PH_3_—S reads was carried out with NOIseq version 2.30.0 where the data is simulated 5 times to generate technical replicates (Tarazona et al. 2015) and were normalized as Trimmed Mean of M values (TMM). The experimental design was set with PH_3_-S strain as the reference and the PH_3_-R strain as contrast condition. Gene set enrichment analyses (GSEA) was performed in OmicsBox using Fisher’s exact test enrichment analysis. GSEA was performed by uploading the annotation file (.b2g) that was downloaded from BIPAA and also the rank list obtained from the “create count table” analysis. Assignment of enzyme codes and Kyoto Encyclopaedia of Genes and Genomes (KEGG) pathway analysis (KEGG licensed to USDA ARS) were also performed with OmicsBox (Kanehisa and Goto 2008).

### Validating Differentially Expressed *S. oryzae* Transcripts Using qRT-PCR

Differential expression of selected mitochondrial and detoxification genes were validated using qRT-PCR using CFX Connect systems (Bio-Rad) with 10 µl reaction mixture containing 2.0 µl cDNA, 6.5 µl SYBR green mix, 0.3 µl of each primer, and 0.9 µl of RNAse-free water. RPS18 was used as a reference gene. The primers used in this study (Supplementary Table S1) were designed with Primer3 web version 4.1.0. The amplification protocol followed: initial denaturation for 2 min at 95 °C, 40 cycles of denaturation at 95 °C for 15 s, annealing at 58 °C for 30 s, and elongation at 72 °C for 30 s. The qRT-PCR analysis consisted of 3 independent biological replicates with 2 technical replicates. Relative expression was verified using the 2^−ΔΔCt^ method (Livak and Schmittgen 2001). The Student’s *t*-test was used to examine the significant differences between the susceptible and resistant populations, and the qRT-PCR data were given as the mean relative expression level SE.

### Identification of Mutations in *dld* Gene

DLD protein sequences retrieved from the RNA-seq data were aligned with susceptible (ALY05704.1) and resistant sequences (ALY05706.1) of *S. oryzae* retrieved from NCBI-GenBank.

### Molecular Docking of DLD Protein with Phosphine Molecule

#### Building 3D structures of DLD protein

The DLD sequences were retrieved from the transcriptomic data of resistant and susceptible strains of *S. oryzae*. The DLD 3D structures of susceptible strain (‘K’ at 141 position) and the resistant strain (‘E’ at 141 position) was modeled using MODELLER software (https://salilab.org/modeller/). The template structure of the protein was identified using the BLASTp program (https://blast.ncbi.nlm.nih.gov/Blast.cgi?PAGE=Proteins). The alignment between the template and the target DLD protein sequences was performed using the ‘Align2D.py’ script in MODELLER 10.1 software. The models were developed with the cofactor Flavin Adenine Dinucleotide (FAD) using the ‘MODEL-Ligand.py’ script (https://salilab.org/modeller/). The developed models of DLD from both strains were validated using the PROCHECK program available in the SAVES ver. 6.0 server. The amino acid residues which were present in the disallowed region of the Ramachandran plot were further taken for loop building and were validated against their geometry using PROCHECK (https://www.ebi.ac.uk/thornton-srv/software/PROCHECK/).

#### Docking with phosphine molecule

The validated 3D structure of DLD from both the strains was taken for molecular docking study using AutoDock 4.0 (https://autodock.scripps.edu/). AutoDock Tools 1.5.6. was used to prepare, run, and analyze the docking results. The side chain hydrogen atoms and Kollman charges were added during the protein preparation step. Similarly, the ligands were prepared by adding Gasteiger charges. The grid maps were generated for the ligand, phosphine, using AutoGrid 4.0. The grid was fixed in the surrounding mutant site at the amino acid position 141. The box size was 40 Å × 40 Å × 40 Å at the center of the 141 position. A total of 100 conformations were generated using the Lamarckian Genetic Algorithm (GA-LS runs) (Huey et al. 2012).

## Results

### Resistance Toward Phosphine

**Fig. 1.**
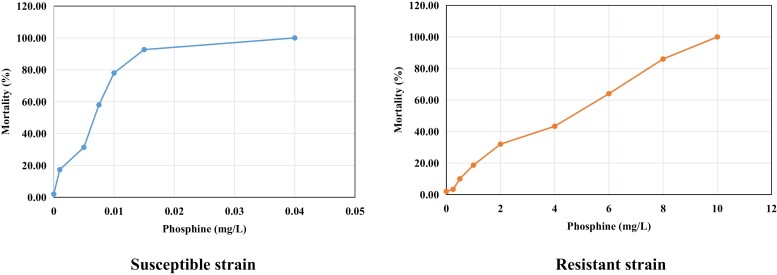
Dose mortality response lines for phosphine susceptible and resistant *Sitophilus oryzae* strains.

In this study, the resistance levels of *S. oryzae* populations collected across the bulk grain storages and the grain supply chain were determined by phosphine bioassay. Resistant and susceptible populations were selected from the 32 populations used in this study. The lowest and highest percentage of resistance were observed in ISO-TNAU-S (0.04 mg L^−1^) and ISO-TNAU-R (0.25 mg L^−1^) populations respectively. The resistant strain was from Pudukottai (Tamil Nadu) harbored high resistance to phosphine with 100.0 and 96.45% at high and low concentrations of phosphine respectively. Whereas, the susceptible strain was from house hold in Trichy grain supply chain that showed 0.00% resistance in both high and low concentrations of phosphine. The LC50 value for phosphine was 3.550 and 0.012 mg/L in resistant and susceptible *S. oryzae* populations respectively. The response curves were different and nonoverlapping fiducial limits were observed at LC50 (0.011–0.013 for susceptible strain and 2.392–5.619 for resistant strain) indicating that these populations were phenotypically different (Table 1, Fig. 1).

**Table 1.** Dose mortality response of resistant and susceptible strain of *S. oryzae*

S. no.	Population	LC_50_ in mg/L (95% fiducial limits)	LC_90_ in mg/L (95% fiducial limits)	Slope ± SEM	*χ*² (df)	Heterogeneity
1	Susceptible	0.012 (0.011–0.013)	0.014 (0.013–0.015)	4.78 **± **1.04	2.65 (3)	0.884
2	Resistant	3.550 (2.392–5.619)	17.252 (10.380–46.164)	1.87 **± **0.14	12.11 (4)	5.027

### RNA Sequencing

A total of 117,868,731 reads were obtained from the susceptible and resistant (both treated and untreated) strains which have been submitted to the NCBI database (Accession number: SRX16017226). After trimming the low quality data, 110,614,950 reads were taken for downstream analysis. Then the sequences were aligned and classified as unique, multi mapping, chimeric, and unmapped reads (Table 2). However, there were no chimeric reads present in both the datasets.

**Table 2.** Illumina paired-end read and STAR alignment statistics

Features	ISO-TNAU-RT strain (treated)	ISO-TNAU-RC strain untreated)	ISO-TNAU-ST strain (treated)	ISO-TNAU-SC strain (untreated)
Total reads	41,621,785	16,222,527	44,259,413	15,765,006
Processed reads	38,459,174	15,786,480	41,280,634	15,088,662
% of high quality data	92.40	97.31	93.26	95.71
Unique reads	28,840,058/82.477%	13,988,193/88.6%	24,076,880/63.76%	11,548,720/76.53%
Multi mapped reads	4,546,101/13.001%	716,545/4.53%	9,779,338/25.897%	749,457/4.96%
Unmapped reads (too short)	1,423,167/4.07%	1,019,807/6.46%	3,783,728/10.02%	2,735,574/18.13%
Chimeric reads	0/0%	0/0%	0/0%	0/0%
GT/AG	17,241,773	9,336,500	15,570,119	6,937,594
GC/AG	78,745	42,725	95,222	31,496
AT/AC	5,474	2,624	9,869	4,471
GC count (%)	39	43	38	35

### Differential Gene Expression and Annotation

**Fig. 2.**
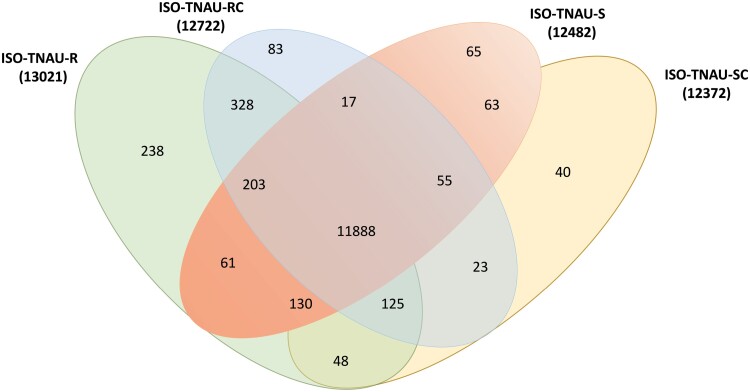
Venn diagram showing the comparisons of number of differentially expressed genes identified with transcriptome data.

After assembling reads, differentially expressed genes were analyzed based on the count table information. A total of 15,057 genes were reported based on the *S. oryzae* reference sequence. In the present study, the total number of genes predicted was observed to be 13,021(ISO-TNAU-RT), 12,722 (ISO-TNAU-RC), 12,482 (ISO-TNAU-ST), and 12,372 (ISO-TNAU-SC). Figure 2 shows the commonly shared gene homologs (11,888) among the 4 strains. Also there were 238 and 65 uniquely expressed genes in ISO-TNAU-RT and ISO-TNAU-ST strains respectively. These genes were mostly with unpredicted functions. Differential gene expression analysis of ISO-TNAU-RT strain in comparison with that of ISO-TNAU-RC showed 3805 differentially expressed genes with probability value of >0.8 in which 1,707 genes were up-regulated (*M* > 0) and 2,098 down-regulated genes (*M* < 0). Whereas in the ISO-TNAU-ST strain, there were 4,235 differentially expressed genes with 2,271 up-regulated genes and 1,964 down-regulated genes. The comparisons of differentially expressed transcripts are tabulated (Table 3) and the list of 10 highly up and down-regulated genes in ISO-TNAU-RT and ISO-TNAU-ST strains are mentioned in Supplementary Table S2a, b respectively.

**Table 3.** Comparison of relative transcripts between resistant and susceptible *S. oryzae* populations (*M* > 0, Probability >  > 0.8)

	Description	ISO-TNAU-RT	ISO-TNAU-RC	M	ISO-TNAU-ST	ISO-TNAU-SC	M
*COX1*	Cytochrome c oxidase subunit I (mitochondrion)	136,486.98	235,523.75	−0.78	162,418.06	442,587.02	−1.44
*COX2*	Cytochrome c oxidase subunit II (mitochondrion)	50,060.89	139,972.64	−1.48	46,916.52	226,651.08	−2.2
*COX3*	Cytochrome c oxidase subunit III (mitochondrion)	64,575.9	132,410.69	−1.03	46,984.04	143,559.01	−1.6
CYTB	Cytochrome b (mitochondrion)	5,658.57	153,379.92	−1.4	22,941.76	111,574.33	−2.2
*ATP6*	ATP synthase F0 subunit 6 (mitochondrion)	22,576.46	89,112.88	−1.9	9,959.96	100,853.9	−3.3
*ATP8*	ATP synthase F0 subunit 8 (mitochondrion)	21.05	116.55	−2.4	0.5	50.65	−6.6
*ND2*	NADH dehydrogenase subunit 2 (mitochondrion)	18,392.53	56,770.96	−1.6	1,903.77	36,694.17	−4.2
*ND3*	NADH dehydrogenase subunit 3 (mitochondrion)	15,437.67	23,461.16	−0.6	8.76	117.03	−3.7
LOC115879491	Dihydrolipoyllysine-residue acetyltransferase component of pyruvate dehydrogenase mitochondrial	1,539.95	2,636.35	−0.7	−	−	−
LOC115875728	Probable pyruvate dehydrogenase E1 component subunit mitochondrial	156.62	476.34	−1.6	−	−	−
LOC115876058	Malate mitochondrial	2,729.79	3,981.77	−0.5	3,349.80	8,206.66	−1.29
LOC115876190	Succinate-ligase [ADP GDP-forming] subunit mitochondrial	109.24	293.91	−1.42	–	–	−
LOC115876274	Fumarate mitochondrial-like	111.21	188.76	−0.76	–	–	–
LOC115876360	2-Oxoglutarate mitochondrial isoform X2	498.83	2,607.21	−2.38	–	–	–
LOC115891126	Succinate dehydrogenase assembly factor 2-mitochondrial	271.13	561.22	−1.04	241.15	420.98	−0.8
LOC115877484	Cytochrome P450 4c3-like	3,027.9	1,089.5	1.4	–	–	–
LOC115875350	Cytochrome P450 306a1	167.81	95.01	0.8	–	–	–
LOC115885021	Glyceraldehyde-3-phosphate dehydrogenase 2	121.09	481.49	−1.99	157.84	241.06	−0.61
LOC115886028	Cytochrome b561 isoform X2	64.49	27.87	1.2	–	–	–
LOC115876404	Cytochrome P450	–	–	–	712.92	110.05	2.69
LOC115885946	Glutathione S-transferase	274.42	141.88	0.9	–	–	–
LOC115888750	Peroxidase isoform X2	5,650.43	2,960.67	0.9	–	–	–
LOC115879483	Venom carboxylesterase-6-like	1,485.98	651.19	1.19	–	–	–
LOC115876621	Carboxylesterase 5A	392.88	188.76	1.05	–	–	–
LOC115881058	Heat shock factor isoform X2	738.38	459.87	0.6	–	–	–
LOC115881911	Acetylcholine receptor subunit alpha-like 2	836.44	357.25	1.2	–	–	–
LOC115885223	Catalase	3,120.7	1,459.43	1.09	–	–	–
LOC115876201	Superoxide dismutase [Cu–Zn]	3,520.0	1,435.29	0.78	–	–	–

M – Log2 ratio of the 2 conditions; Probability – obtained by comparing *M* and difference between 2 conditions (D).

### Differential Expression of Mitochondrial Genes

Analysis on the differentially expressed mitochondrial genes reported the down-regulation of cytochrome c oxidase subunit I (*COX1*) (1.7 folds), *COX2* (2.8 folds), and *COX3* (2.0 folds) in ISO-TNAU-RT strain and respectively. Similarly, NADH dehydrogenase (ND) subunit 2 and *ND3* were down-regulated up to 3.0 and 1.5 folds respectively. Also, the ATP synthase F0 subunit 6 (5.6×) ATP synthase F0 subunit 8 (3.7×) were down-regulated in ISO-TNAU-RT strain. In addition, genes encoding the enzyme dihydrolipoyl dehydrogenase, another name for the phosphine resistance gene linked with *rph2* locus, was up-regulated in resistant strains of *S. oryzae*. However, a decrease in the expression of dihydrolipoyl dehydrogenase was observed in the resistant *S. oryzae* strain treated with phosphine (ISO-TNAU-RT). The orthologous gene Cytochrome b5 desaturase linked with *rph1* locus was significantly up-regulated up to 2.1 folds in ISO-TNAU-RT strain.

The key enzyme in glycolysis viz., glyceraldehyde-3-phosphate dehydrogenase was down-regulated (5.8 folds) whereas the enzyme enolase was up-regulated up to 1.6 folds in phosphine resistant strain (ISO-TNAU-RT).

### Differential Expression of Antioxidant Enzymes in *S. oryzae* Populations

Increase in expression level of superoxide dismutase (up to 2.5 folds) was found in the resistant strain treated with phosphine. Also, the expression of catalase was increased up to 2.1 folds in ISO-TNAU-RT strain. Detoxification genes such as cytochrome P450s (cytochrome P450 306a1 and cytochrome P450 4c3-like), glutathione S-transferase (GSTs), carboxylesterases (venom carboxylesterase-6-like, carboxylesterase 5A) were significantly up-regulated in ISO-TNAU-R strain exposed with phosphine. The uniquely expressed genes such as cathepsin L-like proteinase were down-regulated in resistant *S. oryzae* population treated with phosphine. In addition, there was a decrease in the expression of a novel hemocyte receptor protein croquemort in susceptible strains of *S. oryzae*. Also, many hypothetical genes with unpredicted functions were uniquely expressed in both susceptible and resistant strains of *S. oryzae* (Supplementary Table S3).

The Gene Ontology (GO) analysis of Differential Expression of Genes (DEGs) retrieved 3 ontologies viz., biological process, cellular component, and molecular function. The enrichment analysis also showed the significant down-regulation of mitochondrial function (GO: 0033108, GO: 0009055), response to oxidative stress (GO: 0006979), and oxidation–reduction process (GO: 0055114) (Table 4).

**Table 4.** Gene ontology enrichment analysis of DEGs between phosphine resistant and susceptible *S. oryzae* populations

GO name	GO ID	ES		NES	
		Resistant	Susceptible	Resistant	Susceptible
**Molecular function**					
ATP binding	GO: 0005524	0.54	−0.54	10.07	−7.60
Oxidoreductase activity, acting on the aldehyde or oxogroups of donors, NAD, or NADP as acceptor	GO: 0016620	−0.52	−0.71	−2.82	−4.6
Heme binding	GO: 0020037	0.51	0.50	4.48	2.64
Electron transfer activity	GO: 0009055	−0.57	−0.59	−3.61	−3.22
NAD binding	GO: 0051287	−0.58	−0.57	−2.76	−3.83
NADH dehydrogenase activity	GO: 0008137	−0.51	−0.58	−2.50	−2.69
**Biological process**					
Mitochondrial electron transport, NADH to ubiquinone	GO: 0006120	−0.54	−0.60	−3.30	−3.18
Oxidation-reduction process	GO: 0055114	0.52	0.51	7.36	6.98
Tricarboxylic acid cycle	GO: 0006099	−0.59	−0.76	−4.35	−4.71
Mitochondrial respiratory chain assembly	GO: 0033108	−0.53	−0.64	−2.56	−3.17
Chitin metabolic process	GO: 0006030	−0.50	−0.53	−4.34	−2.65
ATP synthesis coupled proton transport	GO: 0015986	−0.80	–	−4.08	–
Response to oxidative stress	GO: 0006979	−0.61	−0.58	−3.79	−2.85
**Cellular components**					
Mitochondrial respiratory chain complex 1	GO: 0005747	−0.60	−0.63	−4.07	−3.97
Mitochondrial membrane	GO: 0031966	0.54	–	2.68	–
Integral component of organelle membrane	GO: 0031301	0.52	0.51	2.50	2.52
Proton-transporting ATP synthase complex	GO: 0045259	−0.79	−0.86	−3.70	−4.42
Tricarboxylic acid cycle enzyme complex	GO: 0045239	−0.58	–	−0.305	–

ES – enrichment score; NES – normalized enrichment score.

**Fig. 3.**
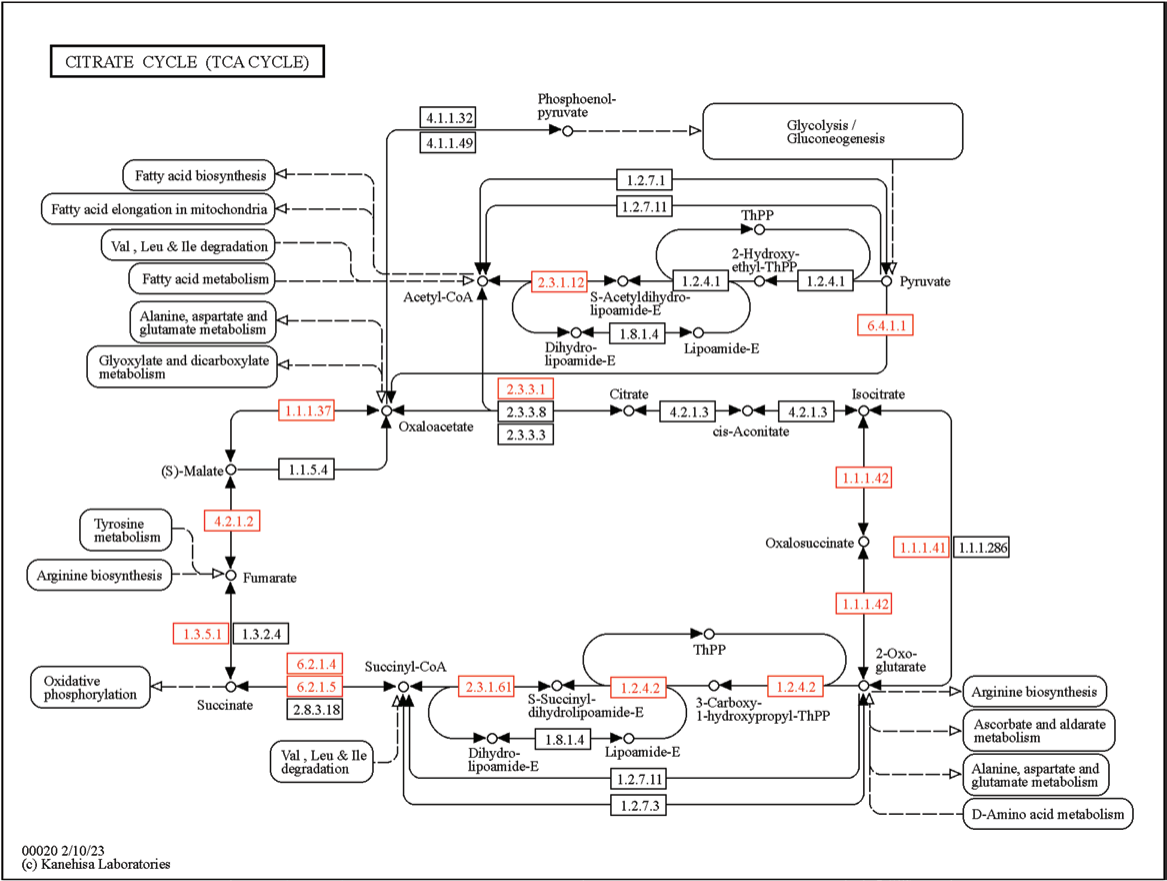
KEGG pathway map showing the differentially expressed (all down-regulated) genes in TCA cycle.

The KEGG analysis of DEG revealed that the genes coding for enzymes involved in TCA cycle were down-regulated whereas the genes coding for glycolysis and metabolism of xenobiotics by cytochrome P450 are enriched (Supplementary Table S4) (Fig. 3). There were 75 metabolic pathways involved in phosphine resistance. The enzymes of the citrate cycle (phosphoenolpyruvate carboxykinase, pyruvate dehydrogenase, pyruvate carboxylase, aconitase hydratase, ATP citrate synthase, succinate dehydrogenase, succinyl-CoA synthetase, isocitrate dehydrogenase, oxoglutarate dehydrogenase), glycolysis (hexokinase, glucose-6-phosphate 1-epimerase, fructose-bisphosphate aldolase, phosphoenolpyruvate carboxykinase, pyruvate dehydrogenase, and alcohol dehydrogenase), and oxidative phosphorylation (succinate dehydrogenase) were mapped in the resistant *S. oryzae* strain that might be involved in the regulation of phosphine resistance.

### Validation Using qRT-PCR

**Fig. 4.**
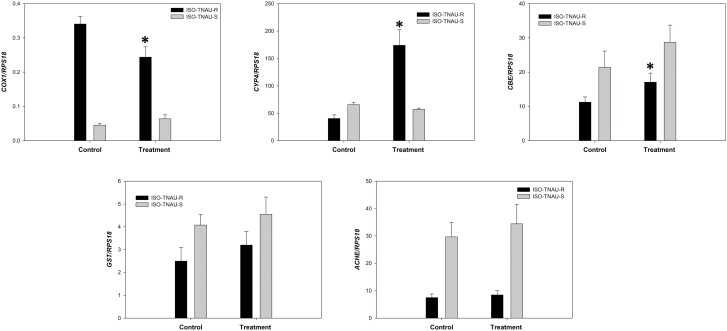
Relative gene expression in susceptible and resistant populations of *S. oryzae*.

The cycle threshold (Ct) value of RPS18 (reference gene) was used to compare the gene expression patterns. Five genes were selected for validation using qRT-PCR. The transcriptome data revealed a significant down-regulation in the expression of the mitochondrial gene *COX1* in the resistant strain treated with phosphine when compared to the untreated (control) resistant strain. The qRT-PCR results correlated with the transcriptome data showing similar target regulation with −1.4-fold for *COX1* gene (Fig. 4). In addition, the up-regulation of the detoxification genes viz., cytochrome P450 4C3 (*Cyp4c*) like, carboxylesterase (*Cbe*), glutathione S-transferase (*Gst*), and acetylcholine receptor alpha-like 2 gene (*Ach*) in the resistant strain treated with phosphine was observed in transcriptome data. Similar expression patterns were observed in the control and phosphine treated resistant strain using qRT-PCR validation with 4.3, 1.5, 1.2, and 1.4-fold changes for *Cyp4c*, *Cbe*, *Gst*, and *Ach*, respectively (Fig. 4).

### Mutants in *dld* Gene of *S. oryzae* Populations

**Fig. 5.**
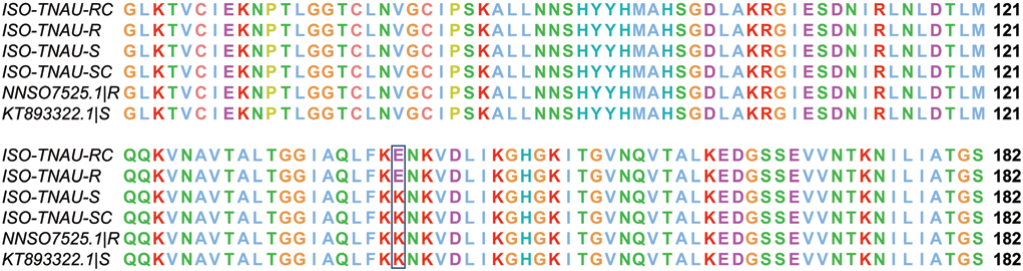
Alignment of dld protein of *S. oryzae* strains. The resistant variant occurs at position 141. Susceptible populations have the amino acid K at this position whereas, the resistant variant has E at the particular position.

Aligning the translations of DLD of all resistant to susceptible sequences revealed a Lysine > Glutamic acid (K141E) substitution Fig. 5 which is located at the nearer to the active site cavity. The K141E variant is found to be homologous to the resistant variant in *R. dominica* K142E (Schlipalius et al. 2012). Unlike the P45S, the K141E mutation is present away from the FAD binding domain and is not involved in FAD cofactor binding.

### Modeling 3D Structure of DLD Proteins

The crystal structure of human DLD (PDB ID: 6I4Z; resolution: 2.34 Å) was identified as a suitable template for modeling the *S. oryzae* DLD protein. This template structure showed 73.4% sequence identity with *S. oryzae* DLD protein. Among the 5 generated models, the best model was chosen based on the DOPE score. Dope score for the resistant and susceptible DLD protein which was chosen in the present investigation was observed to be −1044.15 kcal/mol and 1256.66 kcal/mol, respectively.

Amino acid residues (Ala10, Leu20, Ala26, Ser27, Leu28, Gln30, Arg31, Tyr33, Ser34, Ser36, Asp38, Ala39, Thr72, and Pro485) which occurred in the disallowed region of Ramachandran plot were loop modeled and developed into a valid model of resistant DLD protein with single residue, that is, Ser27 present in the disallowed region of Ramachandran plot (Supplementary Fig. S2a). Similarly, the initial model for the susceptible DLD protein had amino acids such as Thr8, Ala10, Leu20, Ala26, Ser27, Leu29, Arg31, Tyr33, Ser35, Ser36, Asp38, Ala39, Thr72, and Pro485 in the disallowed region of Ramachandran plot (Supplementary Fig. S2b). The final validated model of DLD protein from susceptible strain showed the presence of the residue Leu20 and Cys487 in the disallowed region with the DOPE score of −1256.66 kcal/mol (Supplementary Fig. S3a, b).

### Docking of DLD With Phosphine

**Fig. 6.**
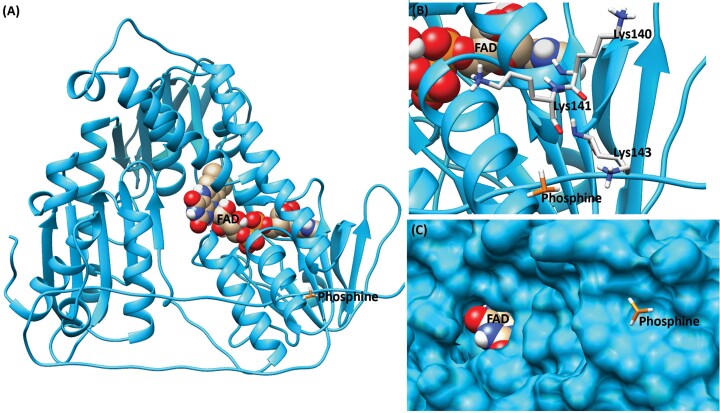
Docked complex of phosphine into susceptible DLD protein. A) Full structural view of the docked complex. DLD protein is represented as a ribbon model, FAD as ball, and stick model with heteroatom coloring (oxygen in red, nitrogen in blue, hydrogen in white, ferrous in orange, and carbon in gold) and phosphine as a stick model. B) Interaction view of the docked complex. Interacting amino acid is represented as stick model with backbone carbon atoms in light gray. C) Surface view of binding pocket with FAD and phosphine molecules.

The docking of phosphine molecules into susceptible DLD protein generated 2 clusters of conformation out of 100 GA-LS runs. The first cluster was formed with 48 conformations with a total mean binding energy of −1.71 kcal/mol, whereas the second cluster was formed with 52 conformations and a total mean binding energy of 1.64 kcal/mol (Table 5). The interaction results of the lowest binding energy conformation (−1.72 kcal/mol) from the cluster ranked 1 were selected for analyzing the binding mechanism of phosphine into DLD protein. The amino acid residues such as Lys140, Lys141, and Lys143 were involved in the van der Waals interaction (Fig. 6).

**Table 5.** Docking results of phosphine into susceptible and resistant DLD proteins

Docked complex	Total number of clusters	Cluster rank	Total number of conformations in the cluster	Run	Mean binding energy (kcal/mol)	Amino acids involved in VDW interaction	Docking energy (kcal/mol)
DLD susceptible–phosphine	2	1	48	76	−1.71	Lys140, Lys141, and Lys143	−1.72
DLD resistant phosphine	2	1	65	70	−1.87	Ala58, Gln59, and Asn142	−1.88

**Fig. 7.**
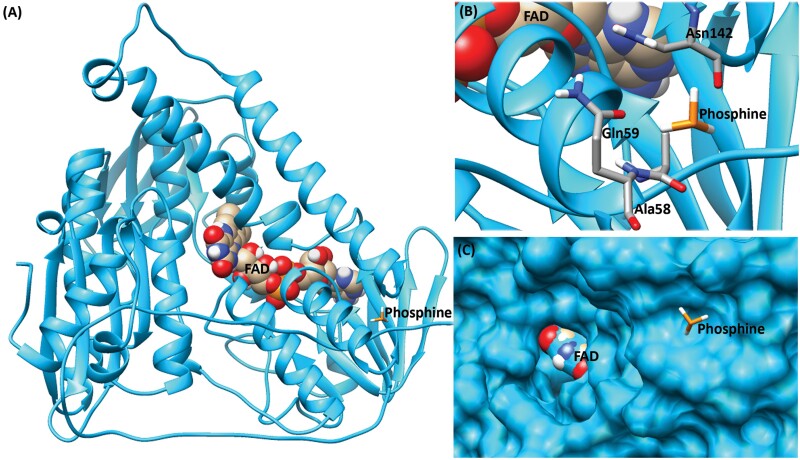
Docked complex of phosphine into resistant DLD protein. A) Full structural view of the docked complex. DLD protein is represented as a ribbon model, FAD as ball, and stick model with heteroatom coloring (oxygen in red, nitrogen in blue, hydrogen in white, ferrous in orange, and carbon in gold) and phosphine as stick model. B) Interaction view of the docked complex. Interacting amino acid is represented as a stick model with backbone carbon atoms in light gray. C) Surface view of binding pocket with FAD and phosphine molecules.

Similarly, molecular docking of resistant DLD protein with phosphine produced a total of 100 conformations with 2 clusters of conformers. Out of 2 clusters, the first cluster showed the presence of 65 conformations with a mean binding energy of −1.87 kcal/mol. Similarly, the second cluster formed with 35 conformations with a mean binding energy of −1.83 kcal/mol (Table 5). The analysis of the binding conformation of low binding energy conformer (−1.88 kcal/mol) from cluster 1 showed that the amino acid residues such as Ala58, Gln59, and Asn142 were involved in van der Waals interaction (Fig. 7). These interaction residues were observed in the scaling factor of 1.00 Å around the interacting ligand molecule.

## Discussion

The emergence of phosphine resistance in stored grain insect pests possesses a serious threat worldwide (Nayak et al. 2020). Strong resistance to phosphine in *S. oryzae* populations from India was reported in 1999 (Rajendran 1999) and from other countries including China, Morocco, Brazil, Australia, and Vietnam (Athié et al. 1998, Zeng 1998, Benhalima et al. 2004, Nguyen et al. 2015, Duong et al. 2016). In this study, high resistance toward phosphine was detected in the Pudukkottai population whereas low resistance in household populations of *S. oryzae* collected from Trichy. The frequency of phosphine resistance observed in the present study is higher than the previous reports from Australia (Holloway et al. 2016), Brazil (Athie and Mills 2005), India (Rajendran 1999, Sivakumar 2009, Rajan et al. 2017), and Turkey (Tings et al. 2018). To establish successful resistance management measures, an attempt was made to understand the molecular variances in resistant insect populations. This study uses gene expression analysis to look at the phosphine resistance mechanism in *S. oryzae*. RNA-seq analysis of phosphine susceptible and resistant populations of *S. oryzae* (treated and untreated) identified differentially expressed genes using the reference transcriptome.

The down-regulation of cytochrome oxidases in ISO-TNAU-RT strain suggested that the inhibition of cytochrome is not directly responsible for the mortality of these insects (Nakakita et al. 1971, Anand et al. 2012). When cytochrome was inhibited by phosphine, H_2_O_2_ was found to be released by intact mitochondria using NADH-linked substrates (Turrens and Boveris 1980, Anand et al. 2012). This hinders the electrons to move along the electron transport chain from NADH dehydrogenase to oxygen which is reduced to superoxide that rapidly dismutates to H_2_O_2_ accumulation thereby leading to mortality (Bolter and Chefurka 1990).

A primary enzyme involved in glycolysis, glyceraldehyde-3-phosphate dehydrogenase, was found to be down-regulated in the phosphine resistant strain. However, the induction of enolase recovers dysfunctional glycolysis due to phosphine toxicity. Enolase was up-regulated in phosphine resistant strain indicating the normal function of glycolysis (Park et al. 2008). Hence, the genes involved in glycolysis could be a potential target for the management of storage grain insect pests as it is a key player of energy production through ATP formation (Bhagavan and Ha 2015).

The phosphine resistant gene, *dld*, was highly up-regulated in resistant populations (ISO-TNAU-RT) of *S. oryzae*. However, a decrease in the expression of *dld* gene was observed in ISO-TNAU-RT strain indicating the lack of interaction between the ferric cluster in *dld* protein and the phosphine radicals (Park et al. 2008). The *dld* gene is a part of the pyruvate dehydrogenase and α-ketoglutarate dehydrogenase enzyme complex. The former is involved in the conversion of pyruvate to acetyl-CoA while the latter converts α-ketoglutarate to succinyl-CoA. Both further proceed with a series of chemical reactions to generate ATP. The down-regulation of *dld* has impacted the expression levels of pyruvate dehydrogenase and α-ketoglutarate dehydrogenase which thereby has reduced the expression levels of the energy source that is, ATP (ATP functional subunits 6 and 8) (Morrison 2021). Hence, it is evident that the metabolic pathways viz., glycolysis and TCA cycle has a potential role in phosphine resistance.

Despite *dld* being linked with phosphine resistance mechanism in insects (Schlipalius et al. 2012), the oxidative stress generated by phosphine resistance is regulated by a number of antioxidant enzymes (Yadav et al. 2020). When a cell is challenged by ROS, the expression of genes producing antioxidant enzymes is generally elevated (Liu et al. 2015) which explains the elevated induction of superoxide dismutase up to 2.5 folds in R strain treated with phosphine. Catalase enzymes have the ability to remove free radicals viz., H_2_O_2_ (Yadav et al. 2020)_._ The increased catalase activity in ISO-TNAU-R strain treated with phosphine indicated that the increase in antioxidant enzyme that prevents oxidative stress where ROS is converted to water (Yadav et al. 2020). Also, elevated expression of the antioxidant enzymes is attributed to the phosphine tolerance in insects (Chaudhry and Price 1992). The inhibition of catalase activity in ISO-TNAU-S strain may be the possible cause of phosphine toxicity to insects as it leads to the accumulation of cytotoxic hydrogen peroxide in insect tissues (Bond and Upitis 1973, Hobbs and Bond 1989, Bolter and Chefurka 1990). Decrease in ROS generation in resistant strain in association with decreased energy metabolism shows how the metabolic suppression enhances tolerance toward phosphine (Nath et al. 2011).

Differential expression of detoxification genes viz., cytochrome P450s (cytochrome P450 306a1 and cytochrome P450 4c3-like), glutathione S-transferase (GSTs), carboxylesterases (venom carboxylesterase-6-like, Carboxylesterase 5A) in resistant strain exposed to phosphine indicated that these detoxification genes also play a role in phosphine resistance (Chen et al. 2021). Huang et al. (2019) reported that the overexpression of *CYP* genes is likely to cause phosphine resistance in *T. castaneum*. The 2 major reasons that contributes to higher levels of *CYPs* due to phosphine exposure could be a result of uncoupling oxidase and monooxygenase processes under increased quantities of activated oxygen species, or it could be a result of indirect detoxification of secondary metabolites due to phosphine exposure (Oppert et al. 2015). Decrease in the expression of the lysosomal cysteine protease family protein Cathepsin L-like in resistant strain indicated the reduced apoptotic response to mitochondrial damage through caspases (Oppert et al. 2015). Expression of Croquemort-like protein, a novel hemocyte receptor that recognizes apoptotic cells in the susceptible strain, indicated its increased response towards apoptosis (Franc et al. 1996).

The DLD variant K141E is homologous to a previously reported resistance variant in the *R. dominica dld* gene (K142E) that is located at the entry to the active site cavity (Schlipalius et al. 2012). The location of this mutation may affect the specificity of the phosphine molecule. Previously, Nguyen et al. (2016) reported a substitution mutation in resistant populations of *S. oryzae* at position 505 (N505T) which is located on the carboxyl terminus of DLD polypeptide. Other mutations that were commonly found in *R. dominica* and *T. castaneum* are P49S and P45S respectively that were widely reported in samples collected from India (Kaur et al. 2015), Turkey (Koҫak et al. 2015), USA (Chen et al. 2015), and Australia (Schlipalius et al. 2012). The DLD protein is characterized with 4 distinct domains consisting of FAD binding domain, NAD binding domain, an active disulfide and homodimer formation domain (Brautigam et al. 2005). The K141E mutation in *S. oryzae* is present in the FAD binding domain indicating that phosphine resistant variants are located in the pore or near the active site of the enzyme (Nguyen et al. 2016).

Three-dimensional structure of *S. oryzae* DLD protein has not been solved by any experimental methods. In our study, the template human DLD showed a higher sequence similarity of 73.4% with the target DLD protein. The RMSD values of 2.70 Å and 2.48 Å were observed for resistance and susceptible DLD proteins with template structure respectively. These RMSD values indicated that the modeled DLD protein structures are highly homologous to the template structure.

The docking results of phosphine into DLD proteins showed that the resistant DLD protein made a stable interaction (−1.88 kcal/mol) when compared to the susceptible DLD protein (−1.72 kcal/mol). Furthermore, docking results also indicated that lysine at position 141 of susceptible DLD protein took part in the binding with the phosphine molecule which was absent in resistant DLD protein due to mutation. On the other hand, K141E substitution might have led to structural changes which may cause reduced affinity to the target (phosphine) emphasizing the importance of K141E mutation in phosphine resistance Although, several in silico studies have been done for other pesticides and the genes involving different classes of cytochrome P450 genes (Julio et al. 2017, Dulbecco et al. 2018, Ghaffar et al. 2020), this is the first study involving the in silico analysis for phosphine with DLD.

## Conclusion

This study shows that the increase in resistance towards phosphine in *S. oryzae* populations poses a serious threat to agricultural commodities in storage. The role of the differentially expressed genes involved in mitochondrial function and detoxification aids in the better understanding of phosphine resistance mechanisms. Many genes from 3 different pathways namely, TCA, glycolysis, and oxidative phosphorylation have shown differences in their expression that are essential for the knock down of energy metabolism necessary to sustain stress conditions. In addition, the in silico docking study demonstrated the reduced binding affinity due to the amino acid substitution in the DLD protein which gives deeper insights on the structure of the *dld* gene and the variants detected could be targeted for the genome editing studies that enables pest management.

## Supplementary Material

iead110_suppl_Supplementary_Figures_S1-S3

iead110_suppl_Supplementary_Tables_S1-S4
