# Supplementary material for: Transcriptome profiling and in silico docking analysis of phosphine resistance in rice weevil, Sitophilus oryzae (Coleoptera: Curculionidae)
Source: J Insect Sci. 2023 Dec 30;23(6):29. doi: 10.1093/jisesa/iead110 (PMC10757423; doi:10.1093/jisesa/iead110)
Supplement: iead110_suppl_Supplementary_Figures_S1-S3 [file iead110_suppl_supplementary_figures_s1-s3.docx]

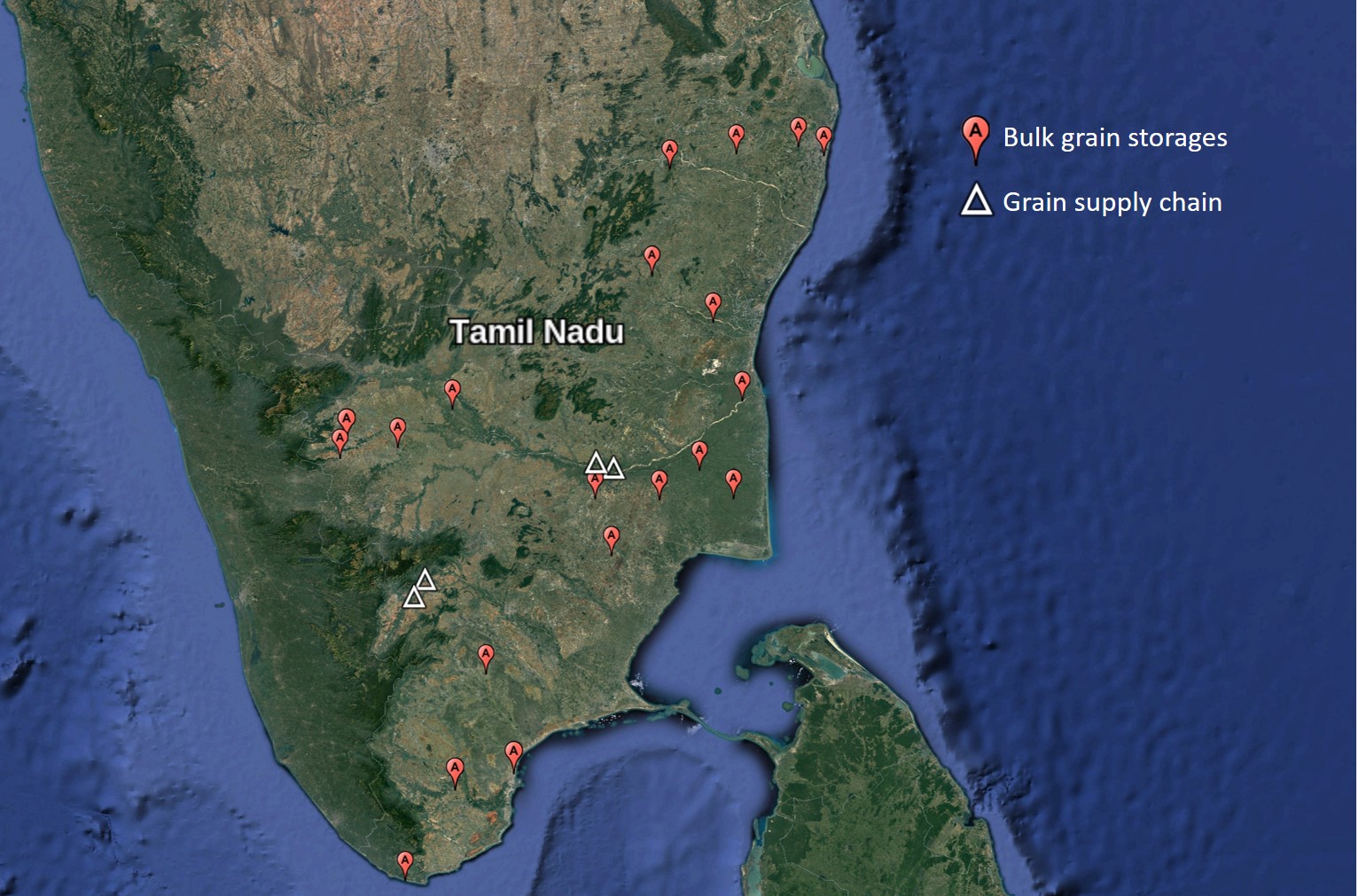


**Supplementary Fig. S1.** Map representing the sampling locations in bulk grain storages and grain supply chains across Tamil Nadu, India


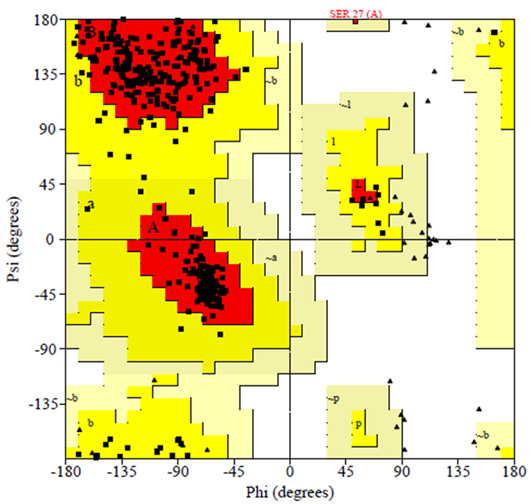


**Supplementary** **Fig. S2a.** Ramachandran plot for the resistant DLD protein

**
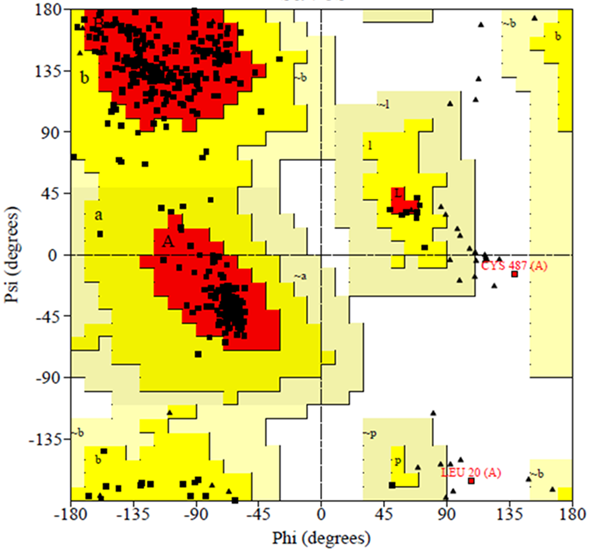
**

**Supplementary** **Fig. S2b.** Ramachandran plot for the susceptible DLD protein

**
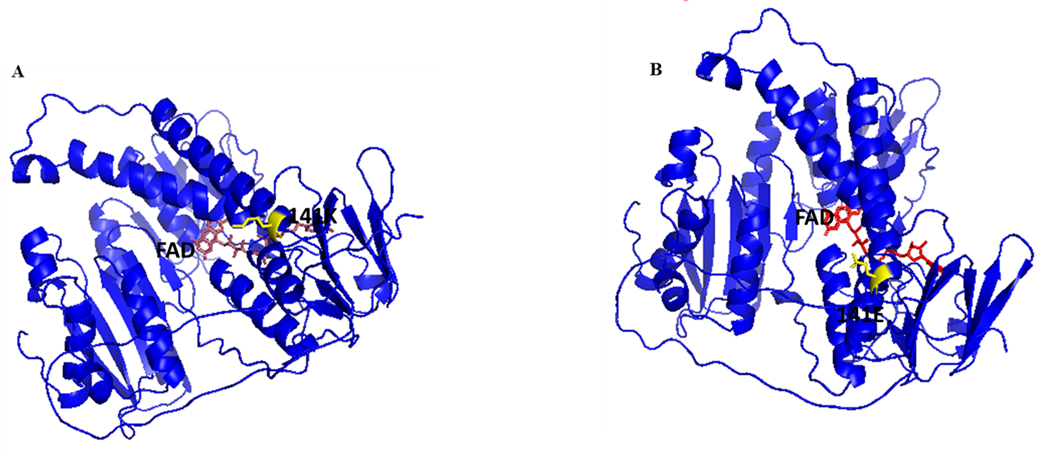
**

**Supplementary** **Fig. S3a, b.** Modelled protein structure of DLD from susceptible and resistant *S. oryzae* strain.

The resistant strain showing mutation at position 141 (K>E)
