# Supplementary material for: Transcriptome profiling and in silico docking analysis of phosphine resistance in rice weevil, Sitophilus oryzae (Coleoptera: Curculionidae)
Source: J Insect Sci. 2023 Dec 30;23(6):29. doi: 10.1093/jisesa/iead110 (PMC10757423; doi:10.1093/jisesa/iead110)
Supplement: iead110_suppl_Supplementary_Tables_S1-S4 [file iead110_suppl_supplementary_tables_s1-s4.docx]

**Supplementary** **Table S1.** List of primers used in this study for qRT-PCR

| **S. No.** | **Gene name** | **Abbreviation** | **Primer Sequence (5)** | |
| --- | --- | --- | --- | --- |
| 1 | Cytochrome c oxidase subunit I | COX1 | F | AAGGAGCTTCTGTTGATCTG |
|  |  |  | R | AGGAGGAGAATAGCAGTGAT |
| 2 | Glutathione S-transferase 1-like | GSt1 | F | ATGGTTCCTTACTCGGACTT |
|  |  |  | R | TAGGCTTTCAATAACGCTTC |
| 3 | Acetylcholineesterase | *Ache* | F | CCGAGGAAGGGTACTACTTT |
|  |  |  | R | TAGGGGTTCAGTTCAGTGAC |
| 4 | Cytochrome P450 4c3-like | Cyp4C3 | F | CGAACGCTGTCTTTCTTAAC |
|  |  |  | R | TGTAGTATCTCAGACCAGCA |
| 5 | Carboxylesterase | Cbe | F | AACTGGCTATACCAACATGG |
|  |  |  | R | TGTCTCCGCTCACTAAATCT |

**Supplementary Table S2a.** List of 10 highly up- regulated transcripts in ISO-TNAU-R strain

| **Sl. No.** | **Locus ID** | **Description** | **M** | **Probability** |
| --- | --- | --- | --- | --- |
| 1. | LOC115882294 | vitellogenin-like | 2.57 | 0.9 |
| 2. | LOC115879935 | apolipophorins | 1.18 | 0.9 |
| 3. | LOC115887947 | peptidyl-prolyl cis-trans isomerase 8-like | 1.19 | 0.9 |
| 4. | LOC115881956 | uncharacterized protein DDB_G0290685-like | 1.65 | 0.9 |
| 5. | LOC115875335 | muscle-specific protein 20 | 1.1 | 0.9 |
| 6. | LOC115875394 | translationally-controlled tumor protein homolog | 1.01 | 0.9 |
| 7. | LOC115884131 | cytochrome P450 4g15-like | 1.96 | 0.9 |
| 8. | LOC115890108 | ribose-phosphate pyrophosphokinase 1 | 1.83 | 0.9 |
| 9. | LOC115877965 | uncharacterized LOC115877965 | 0.67 | 0.8 |
| 10. | LOC115881353 | peptidyl-prolyl cis-trans isomerase | 0.96 | 0.8 |

M- Log2 ratio of the two conditions; Probability- obtained by comparing M and difference between two conditions (D)

**Supplementary** **Table S2b.** List of 10 highly down-regulated transcripts in ISO-TNAU-R strain

| **Sl. No.** | **Locus ID** | **Description** | **M** | **Probability** |
| --- | --- | --- | --- | --- |
| 1. | COX3 | cytochrome c oxidase subunit III (mitochondrion) | -1.03 | 0.9 |
| 2. | CYTB | cytochrome b (mitochondrion) | -1.43 | 0.9 |
| 3. | COX2 | cytochrome c oxidase subunit II (mitochondrion) | -1.48 | 0.9 |
| 4. | ATP6 | ATP synthase F0 subunit 6 (mitochondrion) | -1.98 | 0.9 |
| 5. | ND2 | NADH dehydrogenase subunit 2 (mitochondrion) | -1.62 | 0.9 |
| 6. | LOC115877396 | cathepsin L-like proteinase | -2.06 | 0.9 |
| 7. | ND3 | NADH dehydrogenase subunit 3 (mitochondrion) | -0.6 | 0.8 |
| 8. | LOC115886606 | uncharacterized LOC115886606 | -0.56 | 0.8 |
| 9. | LOC115881949 | alpha-amylase-like | -0.68 | 0.8 |
| 10. | COX1 | cytochrome c oxidase subunit I (mitochondrion) | -0.78 | 0.8 |

M- Log2 ratio of the two conditions; Probability- obtained by comparing M and difference between two conditions (D)

**Supplementary** **Table S3.** Uniquely expressed genes in resistant and susceptible *S. oryzae* strains

|  | **Description** | **ISO-TNAU-RT** | **ISO-TNAU-RC** | **M** | **ISO-TNAU-ST** | **ISO-TNAU-SC** | **M** |
| --- | --- | --- | --- | --- | --- | --- | --- |
| LOC115889314 | uncharacterized LOC115889314 [*Sitophilus oryzae* (rice weevil)] | 1128.63 | 757.58 | 0.5 | - | - | - |
| LOC115884580 | uncharacterized LOC115884580 [*Sitophilus oryzae* (rice weevil)] | 187.55 | 69.67 | 1.4 | - | - | - |
| LOC115875705 | deoxyuridine 5'-triphosphate nucleotidohydrolase-like [*Sitophilus oryzae* (rice weevil)] | 111.87 | 50.67 | 1.1 | - | - | - |
| LOC115883700 | uncharacterized LOC115883700 [*Sitophilus oryzae* (rice weevil)] | 94.10 | 29.13 | 1.6 | - | - | - |
| LOC115890948 | uncharacterized LOC115890948 [*Sitophilus oryzae* (rice weevil) ] | 51.98 | 10.13 | 2.3 | - | - | - |
| LOC115887847 | endoglucanase-like [ *Sitophilus oryzae* (rice weevil)] | 113.19 | 247.03 | -1.12 | - | - | - |
| LOC115883457 | uncharacterized LOC115883457 [*Sitophilus oryzae* (rice weevil) ] | 38.82 | 221.70 | -1.1 | - | - | - |
| LOC115883507 | cathepsin L-like proteinase [*Sitophilus oryzae* (rice weevil)] | 38.82 | 221.70 | -2.5 | - | - | - |
| LOC115887904 | proline-rich receptor-like protein kinase PERK2       [*Sitophilus oryzae* (rice weevil)] | - | - | - | 216.59 | 340.63 | -0.65 |
| LOC115887902 | vegetative cell wall protein gp1-like [ *Sitophilus oryzae* (rice weevil)] | - | - | - | 170.99 | 300.45 | -0.81 |
| LOC115886238 | uncharacterized LOC115886238 [*Sitophilus oryzae* (rice weevil)] | - | - | - | 29.81 | 150.22 | -2.33 |
| LOC115883489 | protein croquemort-like [ *Sitophilus oryzae* (rice weevil)] | - | - | - | 14.03 | 62.88 | -2.16 |

M- Log2 ratio of the two conditions

**Table S4.** KEGG enrichment analysis for differentially expressed genes between phosphine resistant and susceptible populations of *S. oryzae*

| Sequence | Pathway ID | KEGG pathway | Genes annotated to the pathway |
| --- | --- | --- | --- |
| XP_030760844.1 | Map00980 | Metabolism of xenobiotics by cytochrome P450 | LOC115885946 |
| XP_030754475.1 | Map00010 | Glycolysis/Glyconeogenesis | LOC115881205 |
| XP_030760844.1 | Map00982 | Drug metabolism-cytochrome P450 | LOC115885946 |
| XP_030756205.1 | Map00020 | Citrate cycle | LOC115882329 |
| XP_030752244.1 | Map00983 | Drug metabolism | LOC115879484 |
| XP_030747946.1 | Map00630; Map00020 | Glyoxalate and dicarboxylate metabolism; citrate cycle | LOC115876349 |
| XP_030754451.1 | Map00310; Map00380; Map00020 | Lysine degradation; Trypatophan metabolism; Citrate cycle | LOC115881186 |
| XP_030762320.1 | Map00020 | Citrate cycle | LOC115887127 |
